# Global trends in type 1 diabetes in adolescents and young adults (1990–2019)

**DOI:** 10.1038/s41390-024-03554-0

**Published:** 2024-09-18

**Authors:** Jessy Louisa Silfer, Ksenia N. Tonyushkina

**Affiliations:** 1 Case Western Reserve University Medical School, Cleveland, OH USA; 2 Division of Pediatric Endocrinology, Diabetes and Metabolism, Rainbow Babies and Children’s Hospital/CWRU School of Medicine, Cleveland, OH USA

The study by Gong et al. examines the increases in the incidence, prevalence, and burden trends of type 1 diabetes (T1D) in adolescents and young adults over a 30-year span (1990 – 2019) by age, sex, and geographical regions based on the data from the 204 countries and territories included in the Global Burden of Disease (GBD) collaborative.^1^ Burden is defined as a combination of mortality rates and disability adjusted life year (DALY) indexes. The results of this 3-decade study should be interpreted within the milieu of improvements in the diagnosis and treatment of T1D over this period as well as economic and political regional changes to inform further research, policy development, and advocacy to mitigate the disease burden.

In the pediatric population, T1D due to gradual autoimmune destruction of the pancreatic beta cells (stages 1 through 3)^2^ remains a predominant form over type 2 diabetes and other types of diabetes (congenital, MODY, iatrogenic). Also, increasing rates of pediatric obesity are responsible for a dramatic increase in the incidence of youth-onset type 2 diabetes (YOT2D). In the past decades, YOT2D has been frequently treated with insulin and mislabeled as T1D given the lack of other therapeutic options approved for pediatric use. Therefore, total statistics for T1D reported by Gong et al. could include YOT2D cases, particularly in certain geographical regions.

The study by Gong et al. found nearly universal increases in T1D incidence and prevalence in adolescents and young adults between 1990 and 2019 across all age, sex, and socio-demographic index (SDI) groups (Tables 2 & 3, Gong et al.). Such epidemiological changes are multifactorial; genetic predisposition, environmental factors, including diet and infections, and psychosocial factors need to be considered. Interestingly, the relative impact of the genetic component might be reduced in the industrialized world as the frequency of the highest-risk genotypes has decreased since 1978.^3^ The global rates of autoimmune diseases including T1D have steadily increased along with T1D autoimmune comorbidities; environmental factors and infection agents are thought to be implicated.^4^ The increased incidence of childhood obesity has been blamed for accelerating progression to stage 3 T1D in genetically predisposed individuals by several mechanisms. The accelerator hypothesis suggests that proinflammatory adipokine secretion from hypertrophied adipocytes can promote autoimmune inflammation.^5^ Obesity-related insulin resistance, a known risk factor for beta-cell exhaustion, is theorized to contribute to the progression to clinical (stage 3) T1D in individuals with indolent autoimmune beta cell compromise (preclinical stage 1 and stage 2 T1D). The above mechanisms result in double diabetes or type 1.5 diabetes (T1.5D). Challenges of T1.5D, such as a higher likelihood of hyperglycemia, more tedious insulin therapy, and higher complication rates may become more prevalent worldwide. Pediatric research and the endocrine community must continue to explore effective ways to address the obesity epidemic.

Consistent with previous studies, the increase in incidence of T1D leading to increased prevalence is likely driven by continued improvement in the accuracy and timeliness of diagnosis over the 30-year period.^6^ The comparisons of T1D incidence, prevalence, and related mortality between countries with different SDI values point to striking disparities. The countries were divided into the following SDI categories: high, high-middle, middle, low-middle, and low SDI. During the 3-decades, the incidence of T1D significantly increased among all SDI groups, with the greatest average annual percent change (AAPC) occurring in middle SDI countries, followed by those countries with a high-middle SDI, a high SDI, a low-middle SDI, and those with a low SDI, which had the lowest AAPC in T1D incidence (Table 2, Gong et al.). There was a similar thirty-year pattern reported in the AAPC for T1D prevalence (Table 3, Gong et al.). One potential explanation for these data is that the middle SDI countries experienced the most economic and healthcare infrastructure development during the thirty years, leading to improved diagnostics of T1D. Also, an increase in the popularity of Western diets and lifestyles potentially led to increased incidence and prevalence of pediatric obesity, insulin resistance, and YOT2D, and progression to clinical (stage 3) T1D. Interestingly, across the study period, three of the top ten highest T1D incidence countries, Norway, Sweden, and Canada, experienced a stabilization in T1D incidence, while in Finland the incidence has slightly trended downward (Supplementary Table 1, Gong et al.). However, the other six high-incidence countries experienced a statistically significant increase in T1D incidence (Supplementary Table 1, Gong et al.). Low SDI countries experienced a minimal increase in T1D incidence and prevalence from 1990 to 2019 likely because these countries faced the least economic growth, changes in lifestyle, and improvements in diagnostic ability from 1990 to 2019. For instance, the systematic analysis for the GBD study 2021^7^ found that low-income countries, such as those in Sub-Saharan Africa, were likely to have lower prevalence rates, but higher rates of missing prevalent cases of diagnosed and undiagnosed T1D. Lower incidence and prevalence rates in low SDI countries can be due to high rates of misdiagnosis of the patient with another condition, such as an infection, or high rates of death from diabetes-related complications or other diseases before receiving a T1D diagnosis.^8^ The minor increase in T1D incidence seen in low SDI countries could be partly due to increased case detection.^8^ It is predicted that with further economic growth in low and low-middle-income countries, the incidence and prevalence of T1D will increase, necessitating the development of care delivery infrastructure and policies to address the increasing economic and societal burden of T1D.^6^

Global T1D mortality trends according to Gong et al. have changed from increasing during the 1990-1999 period to decreasing from 2000 to 2019 within each SDI group. The greatest AAPC reduction in mortality occurred in high-middle SDI countries, followed by middle SDI, high SDI, and lastly, the low SDI and low-middle SDI countries, which had the least AAPC reduction in T1D mortality (Table 3, Gong et al.). From 1990 to 2019, the DALYs AAPC increased in high SDI countries, trended downward in low-middle SDI countries, and decreased in the remaining SDI categories with the high-middle SDI group experiencing the greatest decrease (Table 2, Gong et al.). Mortality from T1D in adolescents and young adults occur mostly from acute complications including hypo- and hyperglycemia, as well as diabetic ketoacidosis (DKA). The therapeutic approaches to T1D have drastically evolved since results of the Diabetes Control and Complications Trial (DCCT) confirmed the importance of intensive insulin therapy via multiple daily injections to achieve tight blood glucose control in1994.^9^ This discovery led to the development and introduction of insulin pumps and glucose monitoring sensors. Subsequent switch in treatment paradigms and tools has likely contributed to the decrease in mortality and DALY scores both globally and in the countries and regions with access to state-of-the-art care. However, lower SDI countries are benefiting less from such innovations, leading to a life expectancy disparity between individuals with T1D in high and low SDI countries. Modeling by Gregory et al. predicted that in 2021, a ten-year-old person diagnosed with T1D in a low-income country had 13 remaining years to live, but the same ten-year-old person diagnosed in a high-income country would have 65 remaining years of life.^6^ One explanation could be that countries with low and low-middle SDI still face the greatest deficit in healthcare infrastructure needed to address maintenance of T1D care and rising incidence.

With age as a mediator, the GBD study by Gong et al. reported an increase in the incidence and prevalence of T1D among all three age groups. Individuals aged 20-24 experienced the greatest AAPC, followed by 15–19-year-olds, and lastly 10-14-year-olds (Tables 2 &3, Gong et al.). The increased T1D incidence in the oldest age group could be partially attributed to the increased incidence of childhood obesity in certain regions.^10^ The increased prevalence among 20-24-year-olds likely results from enhancements in T1D care in the early 1990s that prevented or delayed acute T1D complications such as DKA and hypoglycemia in youth and adolescents. The effects of such T1D care improvements support the age-related mortality trends reported by Gong et al. The AAPC in mortality trended downward in all age groups and reached statistical significance in those aged 15-19 and 10-14 (Table 3, Gong et al.).

The limitations of the Gong et al. analysis include the use of incomplete or subnational level data sets and data from countries and territories collected for periods less than 30 years duration as indicated by the GBD study design reference.^1^ Therefore, the results reported by Gong et al. may be an incomplete picture of the global changes in T1D incidence, prevalence, and mortality. Gong et al. also excluded countries with missing data from their Joinpoint regression analyses, and used the AAPC measure, a weighted average of the annual percentage change, to describe the Joinpoint regression analyses and demographic trends. Such methodological decisions limit the generalizability of global trends.

In conclusion, Gong et al. along with similar large-scale population studies confirmed global increases in T1D incidence and prevalence, the reasons for which are multifactorial and differ by region and SDI level. Studies predicting future trends through mathematical modeling suggest significant increases in T1D incidence and prevalence to continue in low-income/SDI countries. The same geographical regions are currently less prepared to deal with the burden of this chronic condition given currently higher mortality and DALYs. Multifaceted solutions to address the burden of T1D in youth should be tailored to the available resources and economic and political climate in the regions. Major advances in T1D diagnosis and treatment since the discovery of insulin in 1921 including glucose monitoring and insulin delivery technologies make possible the near-normal life expectancy in individuals with this condition. At the same time, lifesaving insulin is still not globally available given its continued prohibitive costs and the need for refrigeration. Also, automated insulin delivery technologies that promise to drastically improve T1D outcomes are expensive and require diabetes team support for success. Diabetes educational campaigns in lower-income countries have the potential to increase T1D case detection, which will likely lead to higher incidence and prevalence, and hopefully decreased mortality.^8^ Global health advocacy and support of low-resource countries is needed to help mitigate disparities and strive for the best possible outcomes for all youth and adolescents with T1D.
